# Case Report: Glucocorticoid Effect Observation in a Ureteral Urothelial Cancer Patient With ICI-Associated Myocarditis and Multiple Organ Injuries

**DOI:** 10.3389/fimmu.2021.799077

**Published:** 2021-12-15

**Authors:** Xiajun Hu, Yumiao Wei, Xinxin Shuai

**Affiliations:** Department of Cardiology, Union Hospital, Tongji Medical College, Huazhong University of Science and Technology, Wuhan, China

**Keywords:** immune checkpoint inhibitor, myocarditis, myositis, case report, tislelizumab, glucocorticoid therapy

## Abstract

Immune checkpoint inhibitor (ICI)-associated immune-related adverse events (irAEs) are becoming important safety issues worthy of attention despite the exciting therapeutic prospects. The growing development of new ICIs also brings new cases of irAEs, raising more challenges to clinicians. Cardiac injury is rare but life-threatening among diverse organ injuries, and effective interventions are critical for patients. Here, we report a novel programmed cell death protein-1 (PD-1) inhibitor tislelizumab-associated severe myocarditis and myositis accompanied by liver and kidney damage in a ureteral urothelial cancer patient, who was firstly treated by cardiologists because of cardiac symptoms. Due to the lack of experience about ICI-associated irAEs, an initial low-dose (0.5 mg/kg/day) and short-term methylprednisolone therapy was used and found to be ineffective and risky to the patient; then, steroid therapy was modulated to a higher dose (1.5 mg/kg/day) with prolonged time course, and improvement of patient symptoms and laboratory markers were observed quickly and persistently. The patient did not show adverse events under this steroid dosage. This case reports a rare tislelizumab-related myocarditis and multiple organ injuries, which provides valuable experience to cardiologists like us. Early recognition of ICI-associated myocarditis and sufficient dosage and time course of glucocorticoid therapy are critical for severe cases. High-quality clinical evidence about the precise diagnosis and therapy in ICI-associated myocarditis and other organ injuries are necessary to guide our clinical works.

## Introduction

Immune checkpoint inhibitors (ICIs) are one of the most promising therapies for cancer, but increasing numbers of reports about immune-related adverse events (irAEs) have indicated security issues worthy of attention during ICI therapy. IrAEs could cover multiple systems, incidence of cardiac injury is low according to the data from clinical trials, and single drug therapy induces less than 1% of events (1). Clinical manifestation of cardiac toxicity could be myocarditis, pericarditis, arrhythmia, heart failure, and cardiac arrest (2). The risk of disease becomes extremely high once myocarditis occurs. A multicenter study showed 40% death in 131 patients suffering from ICI-related myocarditis (3). The balance of risks and benefits during ICI therapy is of great significance. In recent years, some new ICIs are developed, bringing new reports about irAEs. Here, we received a patient with anti-programmed cell death protein 1 (PD-1) monoclonal antibody tislelizumab-induced multiple organ irAEs including myocarditis, myositis, liver damage, and kidney damage. The patient was admitted to the coronary care unit (CCU) because of initial cardiac symptoms. We present the following case in accordance with the CARE reporting checklist.

## Case Description

**Figure 1:**
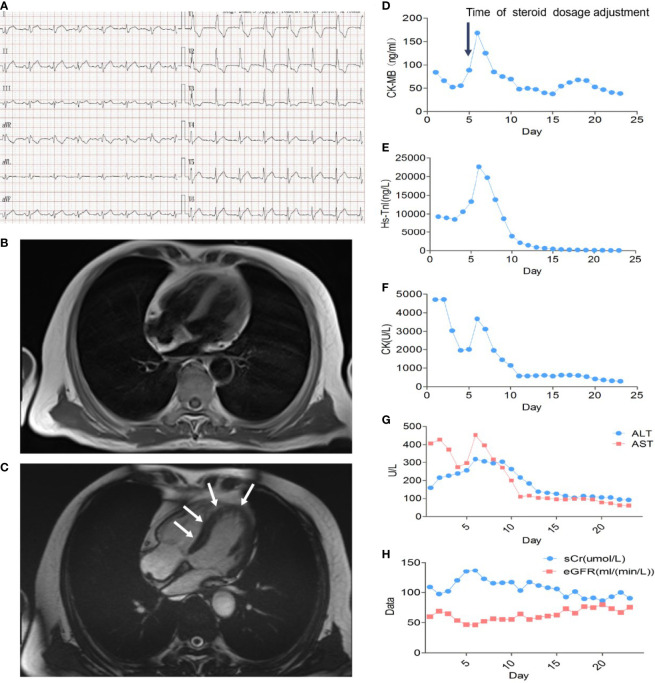
Multiple organ injuries characterized by myocarditis and myositis. **(A)** Electrocardiogram at admission. **(B)** Normal and **(C)** contrast-enhanced imaging in CMR; the white arrow indicated ventricular myocardium LGE. **(D)** CK-MB, **(E)** Hs-TnI, **(F)** CK, **(G)** ALT and AST, and **(H)** sCr and eGFR variation curves during glucocorticoid therapy. The black arrow indicated the time we modulated the dosage of methylprednisolone from 0.5 mg/kg/day to 1.5 mg/kg/day.

**Figure 2:**
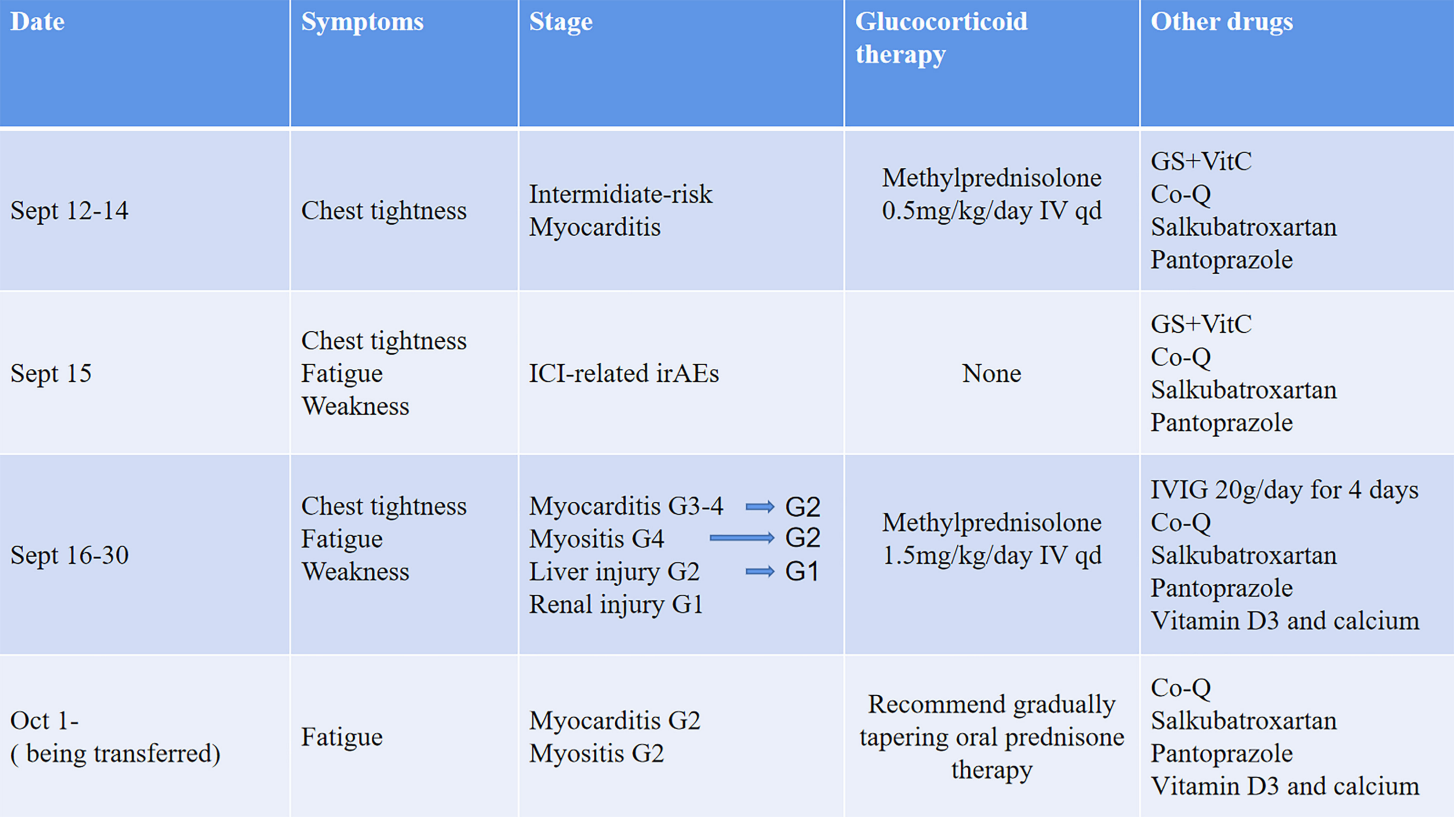
The diagnosis and therapeutic process of the patient. GS, glucose solution; NS, normal saline; Co-Q, coenzyme Q; IVIG, intravenous immunoglobulin. Evaluation of the grade of irAEs is based on *NCCN guidelines insights: management of immunotherapy-related toxicities, version 1.2020* and *Society for Immunotherapy of Cancer (SITC) clinical practice guideline on immune checkpoint inhibitor-related adverse events.*.

**Figure 3:**
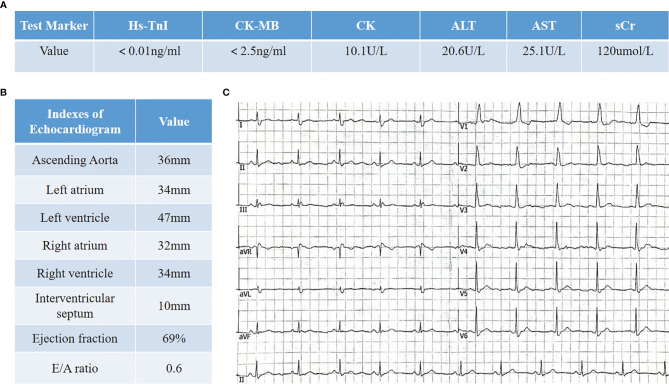
Clinical data of the patient in 1-year follow-up. **(A)** Laboratory indexes involve myocardial zymogram as well as liver and kidney functions. **(B)** Cardiac structure and function evaluation in echocardiogram. **(C)** Electrocardiogram shows CRBBB.

A 66-year-old patient was admitted to the CCU because of persistent chest tightness for 3 days. He had been diagnosed as having ureteral urothelial cancer IV degree and had undergone several courses of radiotherapy and chemotherapy in the Oncology Department. Blood pressure, heart rate, and oxygen saturation of the patient were in the normal range. High sensitive Troponin I (Hs-TnI) was high up to 9,317 ng/L (normal <26.2 ng/L), creatine kinase-myocardial band (CK-MB) was elevated to 84.5 ng/ml (normal <6.6 ng/ml), and N-terminal pro B-type natriuretic peptide (NT-proBNP) level was 291 pg/ml (normal <125 pg/ml). Creatine kinase (CK) value was 4,700 U/L (normal <174 U/L). Aspartate aminotransferase (AST) and alanine aminotransferase (ALT) are greater than 3× the upper limit of normal (ULN). Creatinine level increased to 136 μmol/L (normal <114 μmol/L) with a downregulation of estimated glomerular filtration rate (eGFR) to 46.18 ml/(min/L); urine microprotein including albumin, transferrin, immunoglobulin G (IgG), and α1-microglobulin were detected, indicating acute kidney injury. CD4^+^ T-cell, CD8^+^ T-cell, B-cell, and natural killer cell populations were normal. Serology tests including coronavirus, hepatitis A, B, C virus, human immunodeficiency virus, cytomegalovirus, coxsackie virus, enterovirus, and Epstein–Barr virus were all negative. Positive auto-antibodies against β1 receptor, calcium channel, myosin heavy chain, and ribonucleoprotein indicated an autoimmune response. Coronary artery computer tomography showed slight atherosclerosis. Electrocardiogram (ECG) showed new-onset complete right bundle branch block (CRBBB), atrioventricular block, and intermittent junctional escape rhythm (**Figure 1A**). Cardiac structure and function evaluated by echocardiogram were still in the normal range. Cardiovascular magnetic resonance (CMR) imaging (**Figures 1B, C**) revealed slight multiple segmental myocardium edema and ventricular septum myocardium late gadolinium enhancement (LGE). We first diagnosed the patient as having acute myocarditis and myositis, and gave him a mild dosage of methylprednisolone (0.5 mg/kg/day) intravenously (IV). Symptoms and enzymology improvement were observed, and then methylprednisolone was stopped after 3 days of therapy. However, multiple organ injury-related laboratory indices rapidly rose again (**Figures 1D–H**). Despite chest distress, the patient progressively performed symptoms of cardiac and muscular damage like chest tightness, limb weakness, dorsal myasthenia, diplopia, and dysphagia. We realized that the patient was different from the normal patients we usually treat; autoimmune injuries were quite severe and hard to control in this patient. We further reviewed the patient’s medical history and found that he accepted a single dose of tislelizumab 200 mg IV 20 days ago. An ICI-associated myocarditis and multiple organ injuries were clinically diagnosed. Due to the diverse clinical features and risk of ICI-associated myocarditis, we reference a series of guidelines (4–8) and gave a high dosage of methylprednisolone (1.5 mg/kg) therapy accompanied by intravenous immunoglobulin (IVIG) therapy for the first 4 days. Steroid therapy was prolonged to ensure effective inhibition of autoimmunity induced by tislelizumab. Time axis of diagnosis and therapy of the patient is shown in **Figure 2**. We observed a rapid and persistent improvement of hsTNI, CK-MB, CK, ALT, sCr, and urine microproteins, and gradual recovery of the patient. When hsTNI of the patient fell to 50 ng/L, he was transferred to another medical center for subsequent therapy. The patient accepted outpatient follow-up once after 1 year. Complete symptomatic remission was observed in this patient. No active myocardial or skeletal muscle damage existed according to the laboratory indexes (**Figure 3**). Echocardiogram showed normal cardiac structure and function, except for widening of ascending aorta, which is independent of ICI-associated cardiac injury. Meanwhile, CRBBB consistently existed.

## Discussion

Despite the tremendous efficacy of ICI therapy in cancer, irAEs owing to the growing use of ICIs are becoming major safety issues in cancer immunotherapy. The targets of ICIs, including cytotoxic T lymphocyte antigen 4 (CTLA-4), PD-1, and programmed cell death 1 ligand 1 (PD-L1), all have inhibitory effects on T-cell response, participating in the preservation of immune tolerance and prevention of autoimmunity in normal conditions (9). However, tumors can also evade the immune system by activating these immune checkpoints to develop a tolerable microenvironment to tumor cells. ICIs block the immune checkpoints and accelerate activated T cells to target and kill tumor cells, while the adverse effect of these immunomodulators is that immune tolerance to self-antigens could also be impaired and activated T cells begin to attack any organs of the body (9). The clinical spectrum of irAEs covers broad systems including skin, GI tract, lungs, and endocrine, adrenal, pituitary, musculoskeletal, renal, nervous, hematologic, cardiovascular, and ocular systems (8). The incidence of all adverse events has been estimated to range between 54% and 76% in a meta-analysis (10). The incidence can be increased when combination therapy is used, even reaching 97% (11). The incidence of fatal adverse events is estimated to be between 0.3% and 1.3% (12). Patients suffering from ICI-associated myocarditis have the highest fatality rate. ICI-associated myocarditis is also becoming a new recognized entity, drawing the attention of cardiologists. The risk of myocarditis is significantly higher than other organ injuries; myocarditis accompanied by myositis could be more life-threatening once the muscle injuries involve respiratory and swallowing-related muscles, so early recognition is very important; more aggressive therapies should be considered for those severe cases. Non-oncological clinicians should be vigilant to any suspected cases.

**Figure 4:**
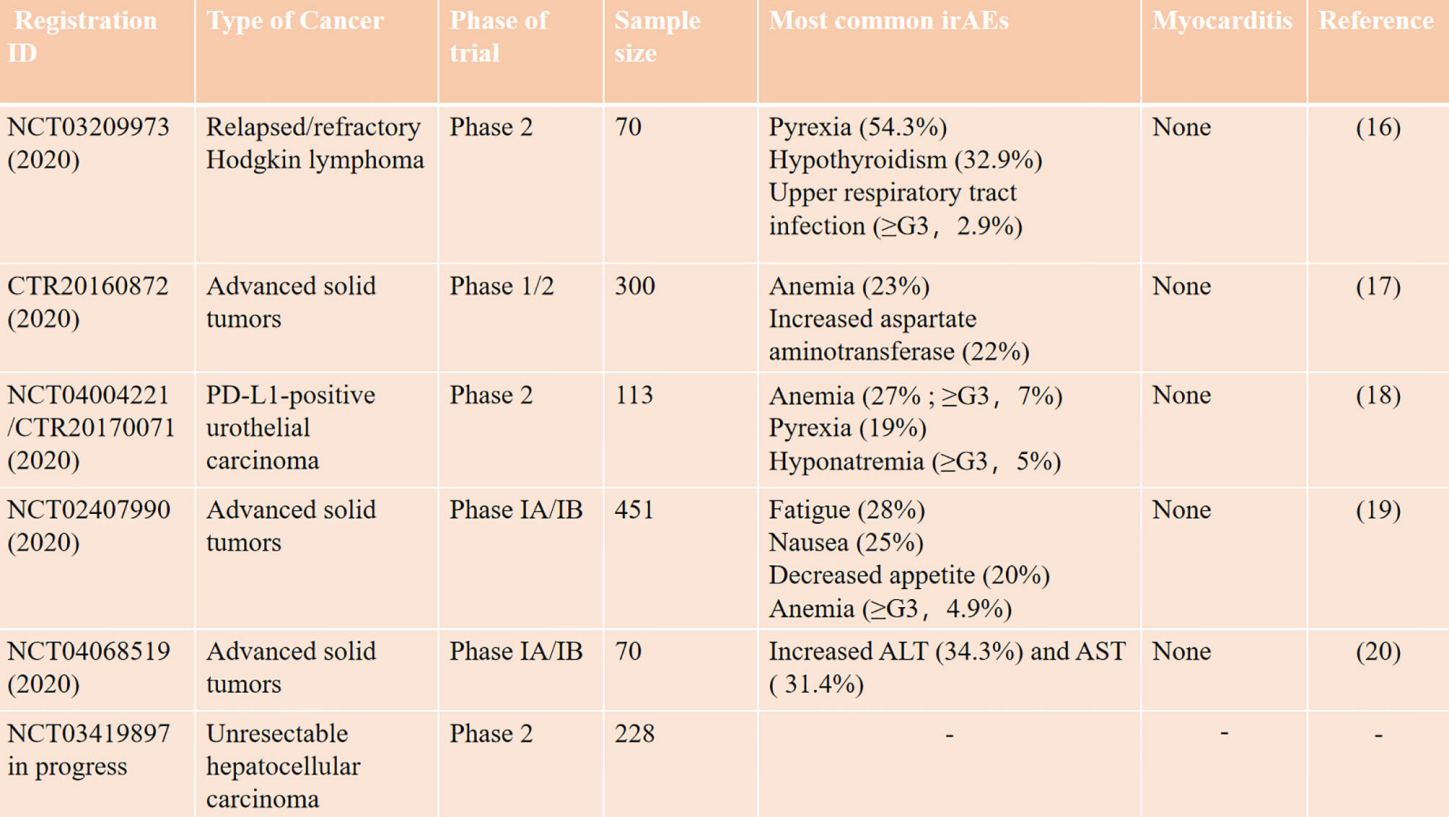
IrAEs of tislelizumab in clinical trials.

Our patient suffered from multiple organ irAEs due to tislelizumab therapy. Tislelizumab is a novel IgG4 monoclonal antibody with high affinity to PD-1 that was engineered to minimize binding to FcγR on macrophages in order to abrogate antibody-dependent cellular phagocytosis (13). Tislelizumab uniquely binds to the CC’ loop of PD-1 with a slow-dissociated rate and completely blocks PD-1/PD-L1 interaction (14). As to ICI-related irAEs, pyrexia and infusion reaction are more specific during tislelizumab treatment (15). A summary about irAEs of tislelizumab in clinical trials is shown in **Figure 4**; myocarditis and myositis are not observed. Only one recent case reported multiple organ injuries following tislelizumab treatment in an advanced non-small cell lung cancer patient (21), while in this case, myocarditis is less severe, and there is not enough reference for us to follow. Our patient presented a rare tislelizumab-related myocarditis, accompanied by myositis and other organ injuries, which also intuitively showed the severity of ICI-associated irAEs to cardiologists like us.

Concerning the diagnosis and therapy, due to the absence of randomized controlled trials (RCTs) and observational studies, there are still no standard diagnostic and therapeutic strategies. ICI medication history is critical. Clinical features of ICI-related myocarditis are similar to acute myocarditis (AM), which is based on symptoms with serum cardiac marker elevation and imageology changes in UCG and CMR. However, a cohort study indicated that LGE was less significant in ICI-associated myocarditis than non-ICI myocarditis (22), and the presence of LGE, LGE pattern, or elevated T2-weighted STIR were not associated with MACE. In our patient, UCG was normal and CMR only showed slight segmental LGE, which is why we underestimated the risk of the patient the first time. More reliable imaging diagnostic strategies are needed for ICI-associated myocarditis.

Corticosteroid therapy is recommended for myocarditis and other irAEs (Grades 2 to 5), but the safe and effective dosage is not precisely pointed out. High-dose glucocorticoids are recommended for severe cases. The ESC guidelines had no exact recommendation for acute active myocarditis due to autoimmunity. Guidelines and consensus of the European Society for Medical Oncology (ESMO) (4), the Society for Immunotherapy of Cancer (SITC) (6), and a recent Expert Consensus Document (7) all recommend prednisone 1–2 mg/kg for severe myocarditis, until the cardiac damage slows down. The National Comprehensive Cancer Network (NCCN) guideline (5) suggests a stronger pulse dosing 1 g/day methylprednisolone for 3–5 days for severe or life-threatening cases, and if cardiac function returns to baseline, the dosage tapers over 4–6 weeks. Meanwhile, recent preclinical trials highlight glucocorticoids’ unexpected effects in cancer therapy like impairing the killing ability of tumor-infiltrating lymphocytes (23) and weakening the effect of ICI therapy (24). Broad adverse effects of glucocorticoid in multiple organs is another concern. Rheumatologists then recommended the concept of glucocorticoid sparing even in severe presentations. Tapering glucocorticoids to the lowest effective dose is recommended within weeks or as soon as improvement is achieved (23, 25). For unstable patients or patients with no improvement after high-dose glucocorticoids therapy, other immunosuppressive agents should be considered, like IVIG, antithymocyte globulin, infliximab, or mycophenolate (4–6, 8), but each with limited supporting data. IVIG can act as an immunomodulator on both innate and adaptive immunity, leading to decreased cardiac inflammation, which was proved to be protective in myocarditis (26). Multiple mechanisms including blocking Fc receptors to interrupt multiple immune cell functions, neutralizing antigens or complement components, and disrupting the apoptosis process may synergistically engage in the immune modulation effect of IVIG. In this case, due to the lack of experiences about ICI-associated myocarditis, we firstly gave a moderate-dose and short-term methylprednisolone therapy. The illness was not controlled and quickly progresses. Then, we tried a high-dose (1.5 mg/kg/day) and prolonged steroid treatment accompanied by short-term IVIG therapy; enzymology rapidly decreased and the symptoms of the patient improved in the following days.

## Conclusion

This is a rare case of myocarditis and myositis associated with a novel PD-1 inhibitor tislelizumab, showing the diversity between ICI-associated myocarditis and common myocarditis. ICI-associated myocarditis and myositis is severe and even life-threatening to patients. Autoimmune damage is significant and persistent, so steroid therapy should be early, given in high doses, and prolonged. We tried a high dosage of steroids with short-term IVIG immunosuppressive therapy in this case, resulting in a positive outcome for the patient. The experience from one case is limited, and high-quality lines of evidence about the precise diagnosis and therapy of ICI-associated irAEs are necessary to guide our clinical works.

## Data Availability Statement

The original contributions presented in the study are included in the article/supplementary material. Further inquiries can be directed to the corresponding author.

## Ethics Statement

Written informed consent was obtained from the individual(s) for the publication of any potentially identifiable images or data included in this article.

## Author Contributions

XH analyzed the patient data, designed the case report, and drafted the manuscript. YW collected the patient data. XS provided significant contributions to the analysis of the patient data and designed the case report. All authors contributed to the article and approved the submitted version.

## Funding

This work was supported by the National Natural Science Foundation of China (Grant No. 31901004 and No. 81600317).

## Conflict of Interest

The authors declare that the research was conducted in the absence of any commercial or financial relationships that could be construed as a potential conflict of interest.

## Publisher’s Note

All claims expressed in this article are solely those of the authors and do not necessarily represent those of their affiliated organizations, or those of the publisher, the editors and the reviewers. Any product that may be evaluated in this article, or claim that may be made by its manufacturer, is not guaranteed or endorsed by the publisher.
